# Acupuncture in patients with trigeminal neuralgia–an exploratory mixed methods trial

**DOI:** 10.3389/fmed.2026.1857785

**Published:** 2026-09-01

**Authors:** Cedric von Angern, Nicolas Volz, Lars Neeb, Barbara Stöckigt, Petra Bäumler, Ina Koubeck, Stephanie Roll, Stefan N. Willich, Benno Brinkhaus, Joanna Dietzel

**Affiliations:** 1 Institute of Social Medicine, Epidemiology and Health Economics, Charité – Universitätsmedizin Berlin, Corporate Member of Freie Universität Berlin and Humboldt-Universität zu Berlin, Berlin, Germany; 2 Department for Neurology and Experimental Neurology, Charité – Universitätsmedizin Berlin, Corporate Member of Freie Universität Berlin and Humboldt-Universität zu Berlin, Berlin, Germany; 3 Institute of General Practice and Interprofessional Care, University Hospital, Tuebingen, Germany; 4 Robert Bosch Center for Integrative Medicine and Health, Bosch Health Campus, Stuttgart, Germany; 5 Department for Anaesthesiology, Multidisciplinary Pain Center, LMU University Hospital, LMU Medizin, Ludwig-Maximilians-Universität München, München, Germany; 6 Neurozentrum Tempelhof, Berlin, Germany; 7 Institute for Biostatistics and Informatics in Medicine and Ageing Research, Rostock University Medical Center, Rostock, Germany

**Keywords:** acupuncture, Chinese medicine, complementary medicine, randomized controlled trial, trigeminal neuralgia

## Abstract

**Background:**

Trigeminal neuralgia (TN) is a chronic pain condition with limited treatment options. Acupuncture has been suggested as a complementary therapy; however, the current scientific evidence is limited. Study aim was to explore feasibility, effects and patient experience of acupuncture for TN in a routine care setting.

**Methods:**

Randomized, open-label, exploratory, multicenter trial with mixed-methods. Patients with TN were randomized to acupuncture plus routine care or to routine care alone. Ten acupuncture sessions were administrated over 8 weeks. The control group received delayed acupuncture using the same protocol after week 12. All patients were followed for 52 weeks. Outcomes were assessed at weeks 8, 12, 20, and 52 including number and intensity of TN pain attacks, Barrow neurological institute Pain Intensity Score, Penn Facial pain, Quality of Life, hospital anxiety and depression, patient global impression of change (PGIC). A qualitative sub-study included six semi-structured patient interviews after completion of treatment, analyzed using content analysis.

**Results:**

Twenty patients (mean age 63.4 years (SD: 15.1; range: 34–85) were included. Both groups showed a reduction in daily attack frequency, not meeting clinical relevance and with no relevant difference between groups (acupuncture: 4.4 (95% CI 1.4, 7.4) control: 4.3 (1.4, 7.1) in week 8), nor was there a relevant difference in daily pain attack intensity between groups (in week 8 mean NRS 3.3 (2.1, 4.5) vs. 3.8 (2.7, 4.9). There was no relevant difference between groups in the BNI or Penn-FPS-R nor the SF-12. Still the acupuncture group experienced greater subjective improvement in the PGIC. Qualitative findings supported the patient’s global impression, with patients describing acupuncture as preventive, calming, and emotionally supportive. The intervention was well accepted, with no serious adverse effects and minor adverse effects such as small hematomas at needling site.

**Conclusion:**

This exploratory mixed-methods trial identified methodological pitfalls relevant to future TN research, including challenges in collecting reliable patient-reported outcomes and accurately documenting paroxysmal pain attacks. Future trials should simplify outcome assessment and reduce participant burden. An acupuncture series was shown to be a feasible, safe, and well-accepted intervention in TN, supporting its further evaluation in adequately powered RCTs.

**Clinical trial registration:**

https://www.drks.de/drks_web/navigate.do?navigationId=trial.HTML&TRIAL_ID=DRKS00018776, identifier DRKS00018776.

## Background

Trigeminal neuralgia (TN) is a debilitating neuralgic pain disorder characterized by recurrent, intense, electric shock-like facial pain. Approximately 90% of patients develop symptoms after the age of 40. The incidence rises steadily with increasing age, ranging from 17.5 cases per 100,000 person-years among individuals aged 60–69 years to 25.6 cases per 100,000 person-years in those older than 70 years (1). The disease often follows a chronic course, severely impacting quality of life. The incidence of TN is 27 cases per 100.000 and the lifetime prevalence was reported to be 0.3% (2, 3). According to the International Classification of Headache Disorders, 3rd edition (ICHD-3), TN is classified into three categories: idiopathic TN, where no structural abnormality is visible on imaging; classic TN, which involves a demonstrable vascular compression of the trigeminal nerve; and secondary (symptomatic) TN, which results from other neurological conditions such as multiple sclerosis, space-occupying lesions, autoimmune or infectious diseases (4).

First-line management typically involves long-term pharmacological therapy using antiepileptic agents such as carbamazepine, valproate, lamotrigine, or gabapentin, often administered in combination (5). However, pharmacotherapy frequently fails to provide sustained pain relief, even at high plasma concentrations. Additionally, the side effect profiles and high interaction potential of these agents present significant challenges, particularly in older adults who commonly take multiple concurrent medications. When pharmacologic therapy is ineffective and a neurovascular conflict is confirmed by imaging, surgical decompression may be pursued. Nevertheless, even surgical approaches have a recurrence rate of approximately 11% (6).

Several systematic reviews of predominantly Chinese trials suggest that acupuncture may be more effective than carbamazepine in reducing pain and attack frequency in trigeminal neuralgia; however, the overall quality of evidence is limited by methodological shortcomings, including the absence of standardized outcome measures, lack of validated questionnaires, and insufficient reporting of attack frequency (7–9). Research from outside China is scarce. To explain acupuncture’s analgesic effects, several neurophysiological mechanisms have been proposed, including activation of polymodal sensory receptors, stimulation of endogenous opioid release (10), and modulation of somato-autonomic reflex pathways (11). There is a need for high-quality, randomized controlled trials and feasibility trials assess the efficacy of acupuncture in this patient population.

Aim of this randomized controlled exploratory trial was to evaluate the effectiveness and feasibility of acupuncture in the treatment of trigeminal neuralgia in a relatively small German population.

## Methods

### Design

**Figure 1:**
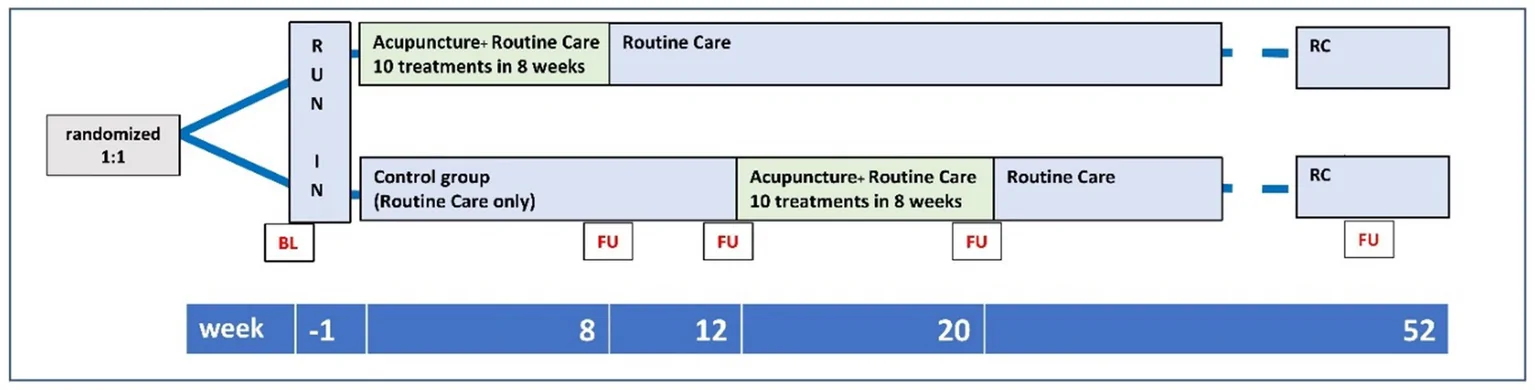
Design of the ACUTRIG Trial. BL, Baseline; FU, Follow-up; RC, Routine care; outcome assessment in week 8, 12, 20, and 52. The six patients for the interviews of the qualitative sub-study were recruited after the end of the acupuncture from both groups.

ACUTRIG was a two-arm, randomized, open-label, exploratory, multicenter clinical mixed-methods trial with parallel group design, conducted in Germany (Figure 1). After randomization, the acupuncture group received 10 sessions over the first 8 weeks alongside routine care. The control group received delayed acupuncture using the same protocol after 12 weeks. All patients received routine care throughout the study period and were followed for 52 weeks, outcomes were assessed at baseline and weeks 8, 12, 20, and 52.

The delayed-entry control design allowed to offer free acupuncture to all participants of the trial and to assess the reproducibility of treatment effects. Recruitment was coordinated through the hospital of the Charité Universitätsmedizin Berlin, supported by three additional Berlin-based practices and an outpatient clinic at Ludwig Maximilian University in Munich.

Randomization followed a 1:1 ratio, stratified by center, using a SAS-generated sequence. Allocation list was concealed to the enrolling physician and accessed by an independent member of the study-team that provided the single allocation on enrollment. The study was open-label; neither physicians nor patients were blinded to group allocation.

The study was conducted in accordance with ICH/GCP guidelines. A vote of the responsible ethics committee (Ethics Committee of the Charité - Universitätsmedizin Berlin) was obtained before the start of the study in September 2019 (Nr. EA1_152_19) and the study was registered in the German registry of clinical trials: DRKS00018776. Patients were recruited until July 2023.

Following amendments were made to the protocol: First, regarding the inclusion criterion “prohibited acupuncture prior to trial participation,” which was increased from a prior four to 12 weeks. Second, we added an evaluation of continuous pain between attacks, and third we extended the trial to a multicenter design to facilitate the recruitment.

### Patients

Inclusion criteria were a diagnosis of idiopathic or classic TN (ICHD-3 criteria), an age of at least 18 and below 85 years, a stable pharmacologic regimen for at least 4 weeks prior to inclusion and a minimum of three daily pain attacks on at least 4 days during the week prior to inclusion with an intensity of at least four on the numeric rating scale (NRS from 0 “no pain” to 10 “most extreme pain imaginable”). The latter inclusion criterion regarding attack frequency and intensity was evaluated by patient diaries during a one-week run-in period prior to randomization.

Exclusion criteria were symptomatic TN, multiple sclerosis, other trigeminal autonomic cephalalgias, recent psychotherapy or acupuncture, opioid or cannabis use, severe somatic or psychiatric illness, limited German proficiency, or concurrent participation in other intervention trials.

Patients were recruited via print and digital outreach, including clinics and social media platforms for trigeminal neuralgia (TN) patients. All patients were informed about the nature and procedures of the study as well as about their right to withdrawal at any time through written patient information sheets and personally during doctor visits. Written and oral informed consent was obtained prior to enrollment.

### Study intervention

**Figure 2:**
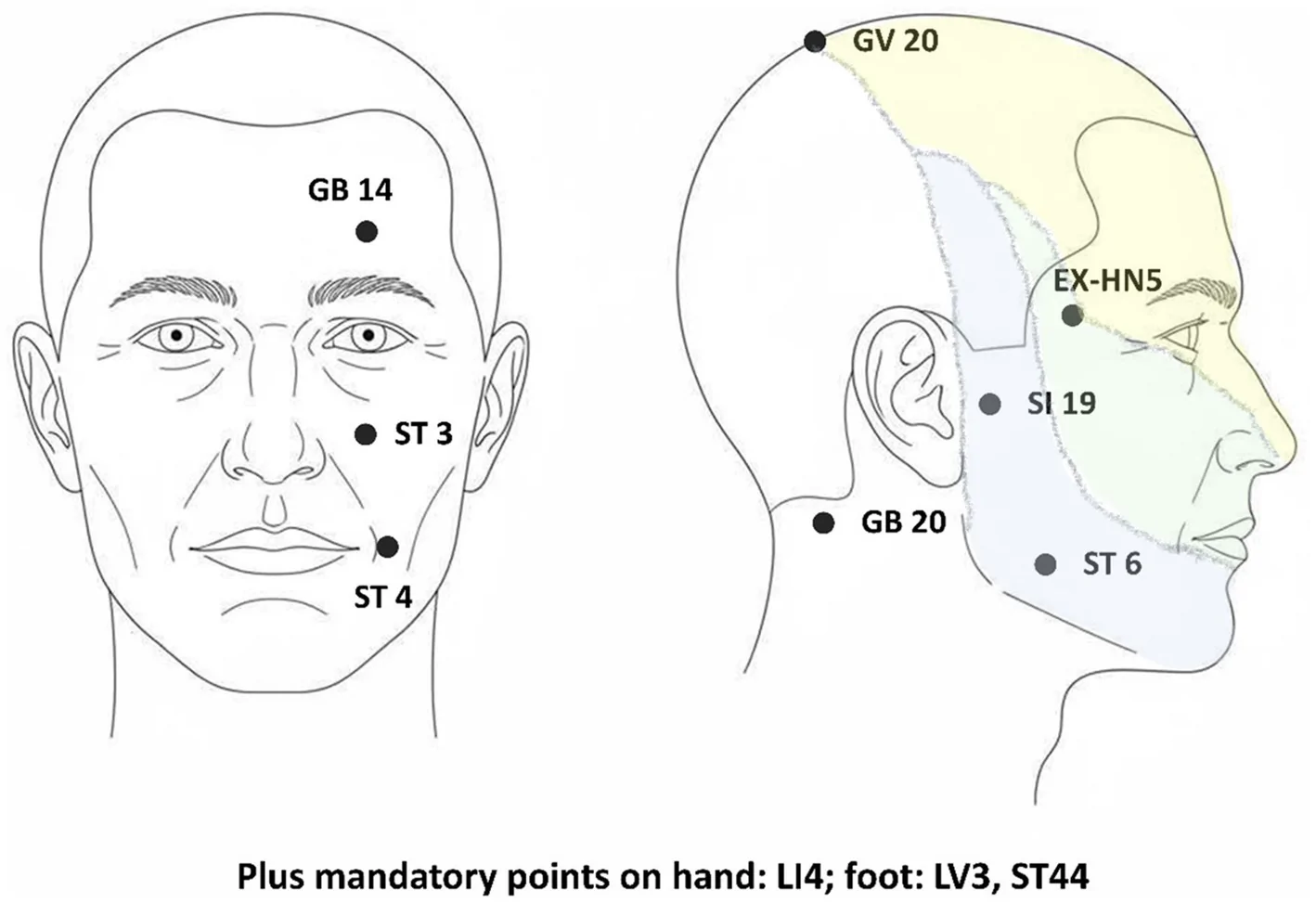
Mandatory points on the face depending on affected trigeminal branch (branch 1 (yellow): GB14, Taiyang––branch 2 (green): ST3, ST4, ST6––branch 3 (blue): SI19, ST4, ST6). Points used contra-lateral at session 4, then ipsilateral only on the affected side.

Ten acupuncture treatments were administered over 8 weeks. Acupuncture treatments were of western medical style an had a semi-standardized regimen according to the affected trigeminal branch and to the diagnosed underlying TCM pattern (wind invasion, stomach heat, yin deficiency, blood stasis). The acupuncture scheme was developed in collaboration with experts from two German physician acupuncture societies, the International Society for Chinese Medicine (Societas Medicinae Sinensis, SMS) and the Medical Society for Acupuncture (Deutsche Ärztegesellschaft für Akupunktur, DÄGfA). In the initial three acupuncture sessions only distal points on hands and feet were used to avoid triggering a pain attack. Starting from the fourth session, contra- and ipsilateral facial points were introduced. Mandatory points were GV20, and bilateral LI4, LV3, ST44, and GB20, with additional facial points tailored to the affected trigeminal branch: VI: GB14, EX-HN5 Taiyang––VII: GB41, ST3, ST4, ST6––VIII: SI19, ST4, ST6 (Figure 2). In addition to the mandatory points, one to four additional facultative points were allowed at the discretion of the acupuncturists. Needles (0.25 × 30 mm) were manually stimulated to evoke the DeQi sensation and were retained for 25 min without further manipulation. All treatments were administered by physicians or advanced trainees with a minimum of 120 h of certified acupuncture training and additional instructions in ICH-GCP and data protection. The acupuncture group received acupuncture directly after randomization from week one to week eight, and the control group received acupuncture from week 12 to week 20.). For more details on acupuncture intervention see Supplementary Table S4 with STRICTA reporting.

Both groups continued medication as part of routine care throughout the study period. Complementary treatments such as Chinese herbal medicine or additional acupuncture were not allowed.

### Outcome parameter

Daily pain attack frequency and pain intensity NRS (0–10) (were assessed via diaries during the week before the intervention (run-in), during the intervention phase (weeks 1–8), during week 12, 20 and 52. Furthermore validated questionnaires were filled out at baseline and at follow-up visits in weeks 8 after the last acupuncture treatment and in week 12, 20, and 52 (Figure 1). Outcomes included the number and intensity of pain attacks, intensity of continuous pain in-between attacks and a set of validated instruments assessing pain, psychological health, and quality of life.

A two-point reduction in the mean pain intensity per week in the diariy entries was considered clinically meaningful (12). A reduction of at least 10% in weekly mean of daily attack frequency was considered clinically meaningful. Participants with a clinically meaningful reduction are considered “responders.” The patient questionnaires administered at baseline and at follow-up visits included the following: Pain intensity was also evaluated by the Barrow Neurological Institute pain intensity score (BNI) a five-level ordinal scale (I: no pain–V: severe pain) (13). The Penn Facial Pain Scale-Revised (Penn-FPS-R) (14), reflected functional interference specific to facial pain by the average score of 12 items each scored from 0 to 10. A reduction of 3.7 points or 75% in the average score is deemed clinically relevant. The Pain Perception Scale (Schmerzempfindungsskala, SES) measures the affect and sensory components of pain perception by two independent sum scores of 14 and 10 items scored from one to four, respectively (15). Symptoms of anxiety and depression were screened by using the Hospital Anxiety and Depression Scale (HADS) (16). Both the anxiety and depression score range from zero to 21 and are composed of seven items scored from zero to three. The minimal clinically important difference has been identified to be 1.7–2 points (12). Health related quality of life is evaluated via the SF-12, a 12-item questionnaire resulting in eight sub-scales and a physical and mental composite score, each with transformed values from zero to 100 (low to high quality of life) (17). The patients’ subjective overall improvement was recorded on Patient Global Impression of Change (PGIC), a 7-point scale for assessing overall change of the general health status from “very much worsened” to “very much improved” (13). Customized questions with 5-point Likert scales assessed perceived changes for daily life at week 8, as well as the expectations of both patients and study physicians regarding the efficacy of acupuncture for existing complaints, as well as their expectations concerning symptom changes related to the study intervention.

Adverse events were assessed at each acupuncture session and could be reported in diaries. Serious events were separately documented and reported within 24 h.

### Statistical analysis

As an exploratory trial aimed at generating preliminary evidence rather than testing confirmatory hypotheses, no formal sample size calculation was performed. A total of 40 patients—20 per treatment group—was considered feasible to recruit within an 18-month period. Patient characteristics were summarized descriptively for the intention-to-treat population (all randomized participants). Continuous variables were summarized as mean (SD), for skewed distributions, median (IQR) is additionally reported. Baseline medication use was described using absolute frequencies. All inferential analyses were conducted using the full analysis set without imputation of missing data. At weeks 8 and 12, group differences in questionnaire outcomes (physical and mental composite scores of the SF-12, Penn-FPS-R, SES affective pain perception, SES sensory pain perception, HADS anxiety, HADS depression) were analyzed using analysis of covariance (ANCOVA), adjusting for the respective baseline value. Daily diary data on attack frequency and attack intensity were aggregated to weekly means per participant. Weekly mean values are presented graphically (mean ± SD by group and week). Group differences at weeks 8 and 12 were analyzed using ANCOVA models adjusted for baseline, with the mean value during the run-in period serving as the baseline measurement.

For all ANCOVA models, assumptions of normally distributed residuals and homoscedasticity were evaluated graphically using residual Q–Q plots and residuals-versus-fitted plots. Results from ANCOVA models are presented as adjusted means per group and adjusted mean differences between groups, with 95% confidence intervals (CI).

At weeks 8 and 12, group differences in ordinal outcomes (PGIC and BNI) were analyzed using ordinal logistic regression models. BNI was analyzed adjusting for the baseline BNI value, whereas PGIC was analyzed without baseline adjustment, as it is a measure of change. Results are presented as odds ratios (OR) with 95% CI.

Additionally, responder analyses were conducted for daily diary outcomes. Responder rates at weeks 8 and 12 were summarized using contingency tables. Group differences in responder rates were analyzed using logistic regression models adjusting for the respective baseline value. Results are presented as OR with 95% CI.

All analyses were exploratory, and no adjustment for multiple testing was applied. Statistical analyses were performed using R (Version 4.5.0; R Core Team, 2025).

### Qualitative sub-study

A qualitative sub-study was conducted in order to further explore patients’ subjective experiences with TN, their medical care and trial participation. The aim was to elucidate further aspects of trial feasibility and experiences of benefit, beyond what the validated questionnaires could assess. Patients were included consecutively in the qualitative sub-study regardless of the study group and interviewed in the weeks after the acupuncture series. Six interviews in total were expected to be sufficient to gather deepened insights within the pilot setting of the study. The interviews were conducted by a team member who was not involved in the intervention.

For the detailed interview guide see Supplementary Table S1. Semi-structured telephone interviews were recorded, pseudonymized and transcribed verbatim. Analysis followed qualitative content methodology using MAXQDA®, with inductive and deductive coding based on interview themes and the research question (18). Categories were developed iteratively to capture emerging insights and to refine analytical depth. The coding process and the results were discussed and reflected in regular team meetings and in a qualitative working group at the Charité. Thereby the quality and intersubjectivity could be enhanced. The qualitative sub-study was supervised by a qualitative expert in the research team and followed the Standards of Reporting Qualitative Research Checklist (19).

## Results

### Patient number and characteristics

**Figure 3:**
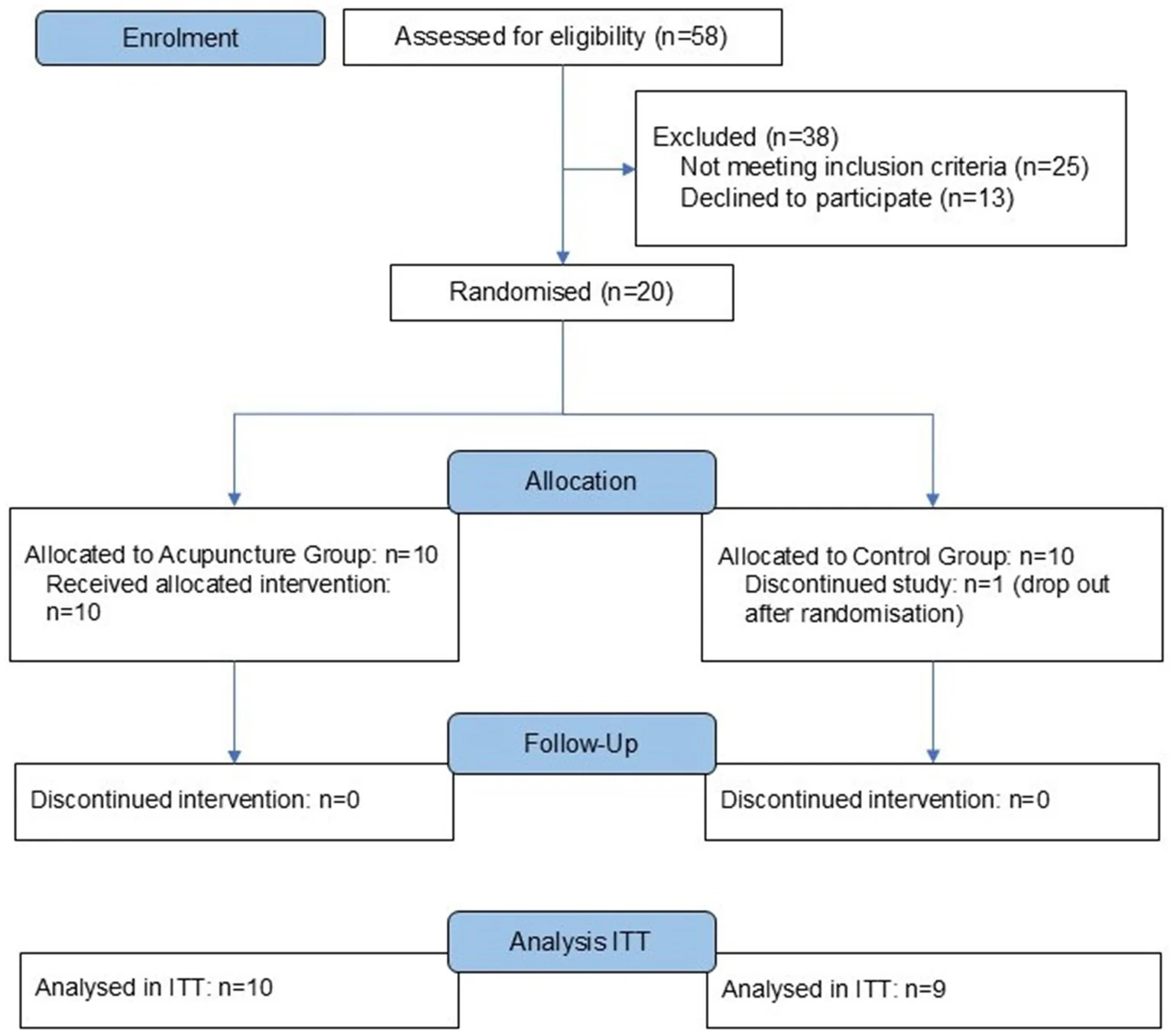
CONSORT flow chart of the ACUTRIG Trial.

Also due to the restrictions during the SARS-VoV2 pandemic only a total of 20 patients were recruited between September 2019 and July 2023. Patients included 14 women and 6 men, with a mean age of 63.4 years (SD: 15; range: 34–85; Table 1). One participant randomized to the control group discontinued before treatment due to dissatisfaction with the allocation, resulting in a final sample of 19 patients available for analyses (Figure 3). The remaining patients came to all planned acupuncture sessions, indicating a good treatment adherence, and completed all follow-up visits.

**Table 1:** Patient characteristics.

Variable	Total (*n* = 20)	Acupuncture (*n* = 10)	Controls (*n* = 10)
Age in years			
Mean (SD)	63.4 (15.1)	63.4 (17.5)	63.4 (13.3)
Median (Q1, Q3)	66.0 (55.5, 75.5)	69.0 (54.0, 79.0)	63.0 (57.0, 73.0)
Gender female, *n*	14	7	7
BMI			
Mean (SD)	24.7 (4.7)	25.7 (5.7)	23.8 (3.3)
Median (Q1, Q3)	24.5 (21.7, 26.5)	24.5 (22.9, 26.5)	24.0 (21.1, 26.6)
Pain attacks number/day, mean (SD)	8.1 (7.4)	8.7 (8.3)	7.5 (6.9)
Intensity of pain attacks on NRS 0–10, mean (SD)	4.7 (2.1)	4.8 (2.1)	4.5 (2.3)
Ongoing medication (gapentinoids, carbamezapin), *n*	20	10	10
TCM syndromes			
Wind invasion	11	6	5
Stomach heat	7	4	3
Yin deficiency	2	0	2
Blood stasis	0	0	0
Symptom duration, *n*			
<1 month	0	0	0
1–6 months	2	1	1
6–12 months	2	2	0
1–2 years	5	3	2
2–5 years	1	0	1
> 5 years	10	4	6
Expectation of acupuncture efficacy physicians *n*			
Very effective	3	0	3
Effective	15	8	7
Slightly effective	2	2	0
Ineffective	0	0	0
Expectations of acupuncture efficacy patients			
Very effective	5	2	3
Effective	13	7	6
Slightly effective	1	1	0
Ineffective	0	0	0

BMI, body mass index; SD, standard deviation. Baseline mean attack frequencies and pain intensities are derived from the 1 week run- in diaries after enrollment.

At baseline, the acupuncture and the control group were comparable in demographics and medication use (Table 1), but regarding the average number of TN attacks and intensity, the intervention group (early acupuncture) showed a higher disease burden. The distribution of trigeminal nerve involvement included V1 in 25% of patients, V2 in 60%, and V3 in 65%. More than one branch of the nerve was affected in 40% of the patients, 30% two branches and 10% all three branches. Half of the patients reported a symptom duration of more than 5 years, and all patients were on pharmacological treatment, partly on combinations; the medications among the patients were Gabapentin (*n* = 12), Carbamazepine (*n* = 11), Oxcarbazepine (*n* = 5), Pregabalin (*n* = 3), and Lamotrigin (*n* = 2), Topiramat (*n* = 1), Phenytoin (v = 1). Three patients had undergone surgery because of their TN. Chinese Medicine (CM) diagnosis by the study physicians revealed wind-cold or wind-heat invasion in 55% of the patients, blazing stomach and liver fire in 35% of the patients (40% in the acupuncture and 30% in the control group), yin deficiency with deficiency heat in 10% of the patients, and blood stasis in 0% of the patients; a combination of syndromes was prevalent within 75% of patients (mostly wind invasion and yin deficiency). Based on clinical judgment, the study physicians expected 14 patients to show a relevant improvement and six to experience a minimal improvement over the course of the acupuncture treatments. Among the patients, five expected the acupuncture to be very effective, 13 expected it to be effective and one expected it to be slightly effective.

### Outcome parameters

**Figure 4:**
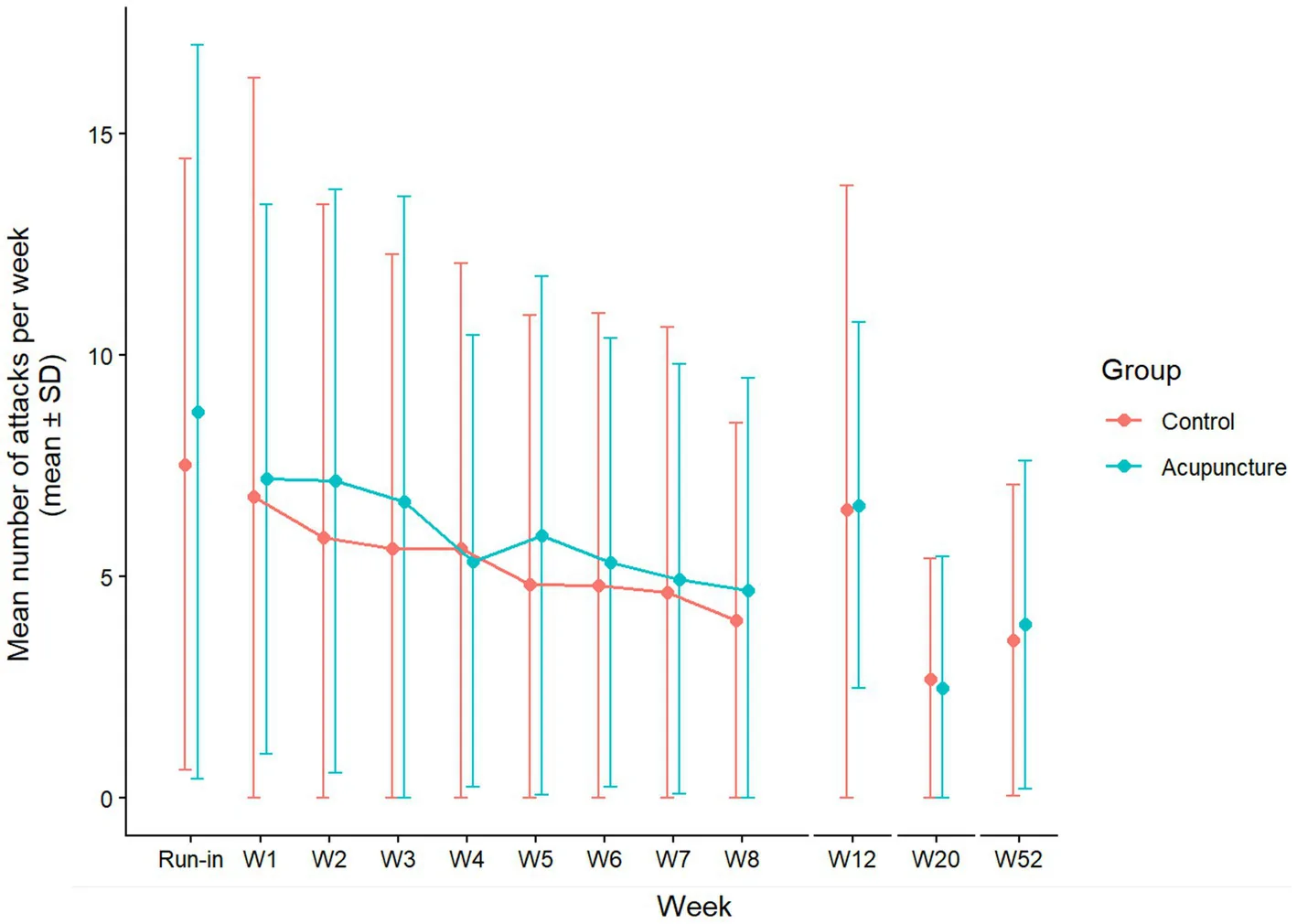
Mean number of daily pain attacks per week (mean ± *SD*). The mean number of pain attacks were calculated for each week of diary entries.

**Figure 5:**
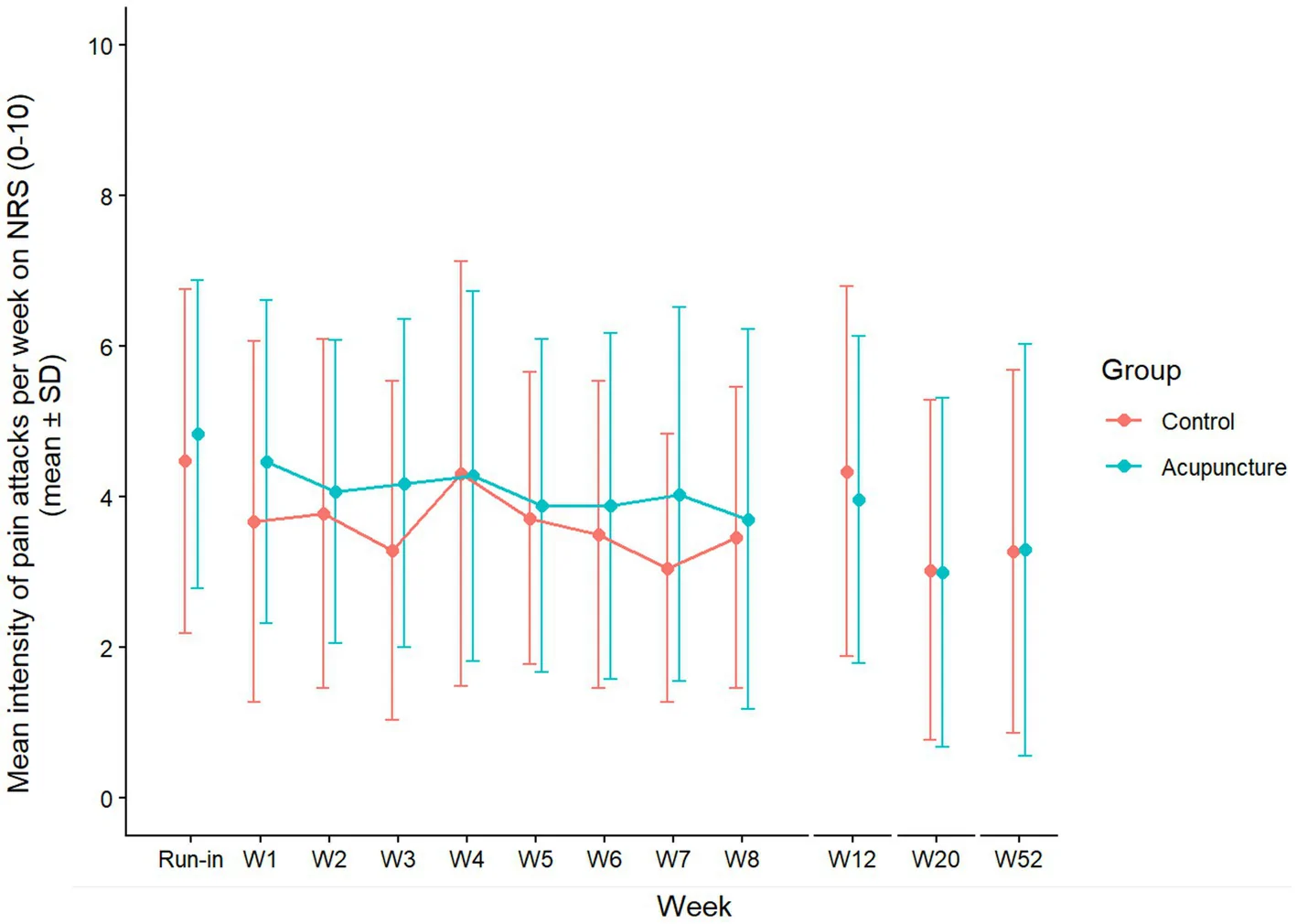
Mean intensity of pain attacks per week on NRS (0–10, mean ± SD) The mean intensity of pain attacks were calculated for each week of diary entries. Pain intensity was rated on a numeric rating scale (NRS- 0 “no pain” 10 “most extreme pain imaginable”).

In week 8 and the early follow up in week 12, both groups showed a relevant reduction in mean daily attack frequency. From run-in the early acupuncture group experienced a reduction in mean daily attack numbers of 8.7 (SD 8.3) to 4.7 (4.8) at week 8 and to 6.6 (4.1) at week 12 and the control group from 7.5 (6.9) to 4.0 (4.5) at week 8 and to 6.5 (7.3) at week 12. There were no relevant difference between groups (baseline adjusted mean (95% CI) in week 8 acupuncture vs. controls: 4.4 (1.4, 7.4) vs. 4.3 (1.4, 7.1) and in week 12 of 5.1 (0.9, 9.4) vs. 7.3 (3.9, 10.8) (Figure 4, Table 2). There was a reduction in intensity of pain attacks (NRS 0–10) from baseline to week 8 from 4.8 (2.1) to 3.7 (2.5) in the early acupuncture group and from 4.5 (2.3) to 3.5 (2.0) in the controls, in week 12 pain intensity was 4.0 (2.2) in the early acupuncture group and 4.3 (2.5) in the controls. The baseline adjusted analysis did not reveal relevant group differences, neither at week 8 nor at week 12 (Table 2, Figure 5). Notably, in the control group, one participant became entirely symptom-free between week 5 and week 7, and another patient reported near-complete remission during the first 8 weeks, which may have disproportionately influenced outcomes in this small sample. Continuous pain was not evaluated because it was not reported in sufficient numbers for evaluation. There were no relevant differences between groups in the BNI or Penn-FPS-R, SES, HADS or the SF-12 (Tables 2, 3, Supplementary Table S2).

**Table 2:** Outcomes adjusted for baseline values in week 8, week 12- before control group received acupuncture.

Score	Acupuncture		Controls		Difference (acupuncture–controls)	
	*n*	Adj. Mean (95% CI)	*n*	Adj. Mean (95% CI)	*n*	Adj. Mean difference (95% CI)
Penn-FPS-R (0 “no pain,” 10 “total inability due to TN pain”)						
Week 8	10	3.1 (1.6, 4.6)	9	4.1 (2.5, 5.6)	19	−1.0 (−3.2, 1.3)
Week 12	10	3.5 (2.0, 5.1)	9	3.9 (2.3, 5.4)	19	−0.3 (−2.6, 2.0)
Attack frequency (number/day)						
Week 8	8	4.4 (1.4, 7.4)	9	4.3 (1.4, 7.1)	17	0.1 (−4.0, 4.3)
Week 12	6	5.1 (0.9, 9.4)	9	7.3 (3.9, 10.8)	15	−2.2 (−7.7, 3.3)
Attack pain intensity NRS (0 “no pain,” 10 “most extreme pain imaginable”)						
Week 8	8	3.3 (2.1, 4.5)	9	3.8 (2.7, 4.9)	17	−0.5 (−2.1, 1.1)
Week 12	9	3.8 (2.6, 5.0)	9	4.5 (3.4, 5.7)	18	−0.7 (−2.4, 0.9)
SES––affective pain perception score (14–56, lower = better)						
Week 8	10	28.5 (22.5, 34.6)	9	31.3 (24.9, 37.6)	19	−2.7 (−11.6, 6.1)
Week 12	10	31.0 (25.4, 36.7)	9	31.0 (25.0, 36.9)	19	0.1 (−8.2, 8.4)
SES––sensory pain perception score (14–40, lower = better)						
Week 8	10	18.3 (16.6, 20.0)	9	20.9 (19.1, 22.7)	19	−2.6 (−5.1, −0.1)
Week 12	10	21.0 (17.2, 24.7)	9	20.8 (16.9, 24.8)	19	0.1 (−5.4, 5.6)
HADS––anxiety score (0–21, lower = better)						
Week 8	10	8.4 (6.8, 10.0)	9	8.7 (7.0, 10.4)	19	−0.3 (−2.6, 2.0)
Week 12	10	9.2 (7.5, 10.9)	9	9.5 (7.7, 11.3)	19	−0.3 (−2.8, 2.2)
HADS––depression score (0–21, lower = better)						
Week 8	9	6.7 (5.1, 8.4)	9	7.7 (6.1, 9.4)	18	−1.0 (−3.4, 1.4)
Week 12	10	6.9 (5.1, 8.7)	9	8.9 (7.0, 10.8)	19	−2.0 (−4.7, 0.6)
SF-12 physical composite score (0–100, <50 indicating worse physical health)						
Week 8	10	44.3 (40.5, 48.1)	9	42.0 (38.0, 46.0)	19	2.3 (−3.3, 7.8)
Week 12	10	47.8 (42.7, 52.8)	9	39.6 (34.3, 44.9)	19	8.2 (0.9, 15.5)
SF-12 mental composite score (0–100, <50 indicating worse mental health)						
Week 8	10	41.9 [36.5, 47.3)	9	39.3 [33.6, 45.0)	19	2.5 (−5.3, 10.4)
Week 12	10	40.8 (33.5, 48.2)	9	39.6 (31.9, 47.3)	19	1.2 (−9.4, 11.9)

Penn-FPS-R, Penn Facial Pain Scale-Revised; SES, Pain Perception Scale (Schmerzempfindungsskala); HADS, Hospital Anxiety and Depression Scale; NRS, numeric rating scale; 95% CI, 95% confidence interval; baseline adjusted estimates derived from analyses of covariance (ANCOVA); Patients in the intervention group received acupuncture from week 1 to week 8. Control group patients received acupuncture from week 12 until week 20.

**Table 3:** Barrow Neurological Institute score for Trigeminal neuralgia (BNI) in week 8 and 12 (post treatment acupuncture group) and in week 20 (post––Treatment controls) and in late follow-up week 52.

BNI score, *n*	Baseline	Week 8	Week 12	Week 20	Week 52
I have no trigeminal pain and do not require medication					
Acupuncture	0	0	0	0	1
Controls	0	0	1	1	1
I occasionally experience pain, but medication is not necessary					
Acupuncture	0	1	0	0	0
Controls	0	0	0	0	1
I experience some pain, but it is adequately controlled by medication					
Acupuncture	4	3	5	7	4
Controls	3	5	4	5	5
I experience some pain, but it is not adequately controlled by medication					
Acupuncture	6	4	3	2	4
Controls	4	2	2	2	2
I experience severe pain and medication does not provide any pain relief					
Acupuncture	0	2	1	1	1
Controls	2	2	2	1	0

Despite the missing difference between both groups regarding pain intensity and attack frequency after 8 weeks, the global impression of change was more positive in the early acupuncture group than in the controls (Table 4). The ordinal logistic regression depicted this trend of higher odds for a poorer global impression of change and a higher BNI-score in the control group but showed no differences between groups: PGIC in week 8 OR (95%CI) 0.20 (0.02, 1.30) *p* = 0.112 and in week 12 of 1.08 (0.21, 5.60) *p* = 0.930 and BNI in week 8 OR (95%CI) 1.69 (0.29, 11.07) *p* = 0.565 and in week 12 of 2.23 (0.31, 19.65) *p* = 0.437.

**Table 4:** Patient global impression of change (PGIC) at week 8 (post-treatment of acupuncture group), week 12 week 20 (post-treatment controls) and week 52.

PGIC score, *n*	Week 8	Week 12	Week 20	Week 52
Very much improved				
Acupuncture	0	0	0	0
Controls	0	1	0	2
Much improved				
Acupuncture	2	1	4	1
Controls	1	1	5	3
Minimally improved				
Acupuncture	6	4	1	4
Controls	3	2	2	3
No change				
Acupuncture	0	3	2	2
Controls	2	3	1	0
Minimally worse				
Acupuncture	0	1	1	2
Controls	2	1	1	1
Much worse				
Acupuncture	1	0	2	1
Controls	0	0	0	0
Very much worse				
Acupuncture	0	1	0	0
Controls	1	1	0	0

Responder analysis did not reveal a difference between groups regarding intensity or frequency of pain attacks between groups in week 8 and week 12 (Supplementary Table S3).

Unadjusted descriptive values of all outcomes over all follow-ups up to week 52 are displayed in Supplementary Table S2.

At week 20, after the control group had received the same acupuncture series as well, a reduction of attack frequency from 6.5 (7.3) in week 12 to 2.7 (2.7) in week 20 and pain intensity from 4.3 (2.5) to 3.0 (2.3) was observed. At the last follow- up in week 52, both groups showed a sustained reduction of less than 50% in attack frequency (Supplementary Table S2).

The information in the diaries regarding changes in medication use over the weeks was unfortunately filled out only sporadically and could not be evaluated.

### Safety

The intervention was well tolerated, with 4 minor adverse effects (one transient increase of attack frequency, one increased urination frequency, one increased bowl movements, one small hematoma), no serious adverse events were reported.

### Qualitative results

Six patients (three men and three women, aged 55 to 85 years) were interviewed 1–4 weeks after the end of their acupuncture series (three starting from baseline, three starting from week 12). Interviews lasted between 14 and 70 min (mean duration 34 min) and were conducted via telephone due to COVID-19 restrictions.

Content analysis revealed the following key domains.

#### Satisfaction with conventional care

Satisfaction with conventional medical care varied. Three patients were satisfied, citing physician expertise and comprehensive care. Others expressed frustration due to limited explanations, lack of empathy, poor communication and insufficient support with medication management, particularly in the context of psychiatric comorbidities.

“With one neurologist, the treatment wasn’t really good because I felt like he could not really handle my despair. And he said straight away that he was a neurologist, not a psychologist. I felt like he could not deal with tears, and he did not let me finish talking.”

(Control group, ACUTRIG_Interview_108, Pos. 46).

All 6 patients expressed a desire for acupuncture to remain part of their ongoing treatment. Most were willing to self-fund the sessions despite financial strain, except one participant who cited financial barriers.

#### Expectations towards acupuncture

Expectations towards acupuncture ranged from mild pain relief to complete avoidance of medication or surgery. Four patients reported that their expectations were met, while two said their hopes for full pain relief without medication were not fulfilled.

“Yes, my expectations were fulfilled. Except that I’m not completely pain-free.”

(Intervention group, ACUTRIG_Interview_501_pseudonymized, Pos. 54).

#### Treatment effects of acupuncture

Five patients reported subjective improvement in symptoms through acupuncture, describing it as prophylactic in nature, reducing both the frequency and intensity of pain attacks. Two mentioned reduced anxiety related to pain episodes, and one noted an improvement in comorbid depression. Two patients (1 control group and 1 intervention group) were able to lower their medication dosage during the acupuncture series. Two patients (both intervention group) described a higher threshold for triggers of pain attacks through mechanical stimuli (cloth stroking the face or tongue touching the lips).

“I definitely had trust […] and nothing could happen to me. It definitely had a soothing effect or prevented relapses. And that did me good.”

(Control group, ACUTRIG_Interview_105, Pos. 34).

One patient (control group) did not perceive lasting benefits and attributed temporary pain relief to symptom fluctuation rather than acupuncture. He reported a pain-free period lasting over a week after the last acupuncture treatment. After that, the pain returned.

#### Perception of the study

Feedback on the questionnaires was heterogenous. While some patients found them manageable or even useful for self-observation, others - particularly older patients - struggled with the complexity or the lack of nuance in response options. Several noted difficulties in recording fluctuating symptoms and suggested the addition of free-text fields.

“I could not really count them—those stabs came really often. One day I even took a piece of paper with me after telling my friend, and she said, ‘Take a paper and make a tally each time.’ And suddenly, I had a lot more attacks than before […]. But I often forget, as soon as the pain is gone.”

(Intervention group, ACUTRIG_Interview_108, Pos. 80).

“I can just say that the questionnaire was too general for me. A text field would have been nice, where one could briefly explain why one answered the way they did.”

(Control group, ACUTRIG_Interview_501, Pos. 64).

## Discussion

### Summary of findings

This exploratory randomized controlled pilot trial examined the feasibility, effects, and patient experience of acupuncture for trigeminal neuralgia (TN). Our findings demonstrate that acupuncture is feasible, safe, and well accepted, while also highlighting the methodological challenges of conducting clinical trials in TN. In this trial, both groups experienced reductions in pain attack frequency and intensity over time, adding acupuncture to routine care did not result in superior quantitative outcomes. In contrast, five of the six participants interviewed in the qualitative substudy reported subjective pain relief and all expressed a desire to continue acupuncture treatment. Regarding the feasibility our findings identified several methodological challenges relevant to future TN research. Older participants frequently reported difficulties completing questionnaires, while the paroxysmal nature of TN made it difficult to accurately record attack frequency, distinguish between individual paroxysms and clusters of attacks, and document changes in concomitant medication. Future trials should simplify data collection, reduce participant burden, and provide closer monitoring of diary completion to improve data quality.

### Strengths and limitations

This study offers several strengths, including its mixed-methods design, a long-term 52-week follow-up, and the use of validated patient-reported outcome measures along with a daily documentation of the attack count in addition to qualitative interviews where subjective experiences could be expressed and quantitative data could be extended. The acupuncture protocol was developed by experienced practitioners and implemented as an individualized treatment within a semi-standardized framework, enhancing both clinical relevance and reproducibility and is reported in line with the STRICTA guidelines. The delayed-entry design allowed all participants to receive acupuncture, which likely improved participant satisfaction and ethical acceptability. This approach enabled the collection of data on the reproducibility and durability of treatment effects across time.

However, several limitations must be acknowledged. As an exploratory trial, the study was not powered to evaluate efficacy, and its results should be interpreted with caution. Recruitment was slower than anticipated—partly due to COVID-19-related restrictions, but also because a high rate of comorbidities excluded many otherwise interested individuals. The small final sample size further limits statistical power. Baseline imbalances, such as a greater number of pain attacks in the acupuncture group, carry risk of bias in such a small sample. Additionally, the preciseness of estimates obtained by the baseline adjusted analyses was poor, due to the small sample size. Furthermore, several participants, particularly older individuals, reported difficulties completing questionnaires and tracking attack frequency in diaries. Similar challenges have been documented in other trials involving older populations (20), suggesting a need to simplify data collection methods in future research. The challenges to keep track of medication logs f.e. led to insufficient data quality in some outcomes with many missings, that cannot be imputated in such small sample sizes. The open-label design with no blinding of patients, acupuncturists, or outcome assessors, combined with mostly patient-reported subjective core outcomes, brings high risk of placebo effect and expectation bias, given the high positive expectations of acupuncture in both patients and physicians. This directly explains the contradiction between subjective benefits in qualitative interviews and negative quantitative objective outcomes.

### Our results in context

Our trial is the first trial exploring feasibility in a central European context, the use of TCM pattern diagnosis and semi-standardized treatment in our study mirrors local clinical practice. A Brazilian three-arm, sham controlled RCT evaluated the effects of add-on: acupuncture or sham acupuncture versus carbamazepine alone in 60 TN patients with trigeminal neuralgia (21). Compared to our trial, an attack count was missing, but rigorous objective outcomes were part of the assessment. A comparable amount of acupuncture sessions was applied in a comparable clinical scenario like ours, which led to a reduction of pain on the VAS in the acupuncture group (*p* < 0.05), but failed to meet thresholds for clinical relevance with a VAS mean difference of less than 10 mm pre and post, and between groups (22). These results generate questions regarding the “dose” of the acupuncture intervention. A recently published, four-armed RCT from China with good methodology compared electroacupuncture (EA) plus carbamazepine (CBZ), sham EA plus CBZ, EA plus placebo, and sham EA plus placebo in 120 TN patients (23). An unusual long 60 min EA three times per week over 4 weeks was administered. This trial had pain attack counting included and could show clinically relevant and sustained improvements in pain intensity and pain attack count for EA plus CBZ with more than 30 mm VAS pain reduction between groups.

### Implications for future research

Given the observed challenges in older patients, future trials should simplify data collection by minimizing the number of questionnaires. According to qualitative data, anxiety of recurring attacks was reduced in our sample, so future trials might want to use an adequate questionnaire to capture this fear. Continued integration of qualitative methods is recommended to ensure that patient experiences and preferences are meaningfully captured.

## Conclusion

This exploratory mixed-methods trial identified methodological pitfalls relevant to future TN research, including challenges in collecting reliable patient-reported outcomes and accurately documenting paroxysmal pain attacks. Future trials should simplify outcome assessment and reduce participant burden. An acupuncture series was shown to be a feasible, safe, and well-accepted intervention in TN, supporting its further evaluation in adequately powered RCTs.

## Data Availability

The raw data supporting the conclusions of this article will be made available by the authors, without undue reservation.
