# Supplementary material for: Acupuncture in patients with trigeminal neuralgia–an exploratory mixed methods trial
Source: Front Med (Lausanne). 2026 Sep 1;13:1857785. doi: 10.3389/fmed.2026.1857785 (PMC13574656; doi:10.3389/fmed.2026.1857785)
Supplement: Supplementary file 1 [file Data_Sheet_1.pdf]

Supplement

Table S1: Interview guide for the qualitative sub-study

**Experience of disease**	**Can you give examples of how TN impairs your daily life? Are there triggers?**<br>**In what phase of your life have you been, when the TN started?**
**Conventional care**	**How did you experience conventional care regarding the TN?**
**Acupuncture**	**Please give an example of how the acupuncture treatment affected your TN.**<br>**How pleasant or unpleasant was the experience of the treatment?**<br>**Are there any other effects of the treatments on your daily life?**<br>**Can you imagine to pay for acupuncture yourself in the future?**
**Trial participation**	**What were your expectations when you started trial participation?**<br>**How easy was it to fill out the questionnaires?**

Table S2: Descriptive values of outcomes over all time points

	Acupuncture (acupuncture between week 1-8)		Controls (acupuncture from week 12 on)	
	**n**	**Mean (SD)**	**n**	**Mean (SD)**
**Attack frequency (number/day)**				
Baseline	9	8.7 (8.3)	9	7.5 (6.9)
Week 8	8	4.7 (4.8)	9	4.0 (4.5)
Week 12	7	6.6 (4.1)	9	6.5 (7.3)
Week 20	7	2.5 (3.0)	9	2.7 (2.7)
Week 52	8	3.9 (3.7)	9	3.6 (3.5)
**Attack pain intensity NRS (0 "no pain", 10 "most extreme pain imaginable")**				
Baseline	10	4.8 (2.1)	9	4.5 (2.3)
Week 8	8	3.7 (2.5)	9	3.5 (2.0)
Week 12	9	4.0 (2.2)	9	4.3 (2.5)
Week 20	10	3.0 (2.3)	8	3.0 (2.3)
Week 52	10	3.3 (2.7)	9	3.3 (2.4)
**SES- pain perception scale affective (0-30, higher indicating worse pain perception)**				
Baseline	10	34.4 (12.3)	9	38.2 (6.7)
Week 8	10	27.3 (12.7)	9	32.7 (8.8)
Week 12	10	29.8 (11.9)	9	32.3 (9.0)
Week 20	10	24.7 (12.4)	9	25.3 (7.4)
Week 52	10	27.1 (11.9)	9	27.0 (12.0)
**SES- pain perception scale sensitivity (0-42, higher indicating worse pain perception)**				
Baseline	10	23.9 (4.9)	9	25.0 (4.7)
Week 8	10	18.0 (3.8)	9	21.2 (3.3)
Week 12	10	20.5 (5.9)	9	21.3 (7.6)
Week 20	10	17.9 (7.0)	9	19.0 (5.9)
Week 52	10	19.0 (8.5)	9	18.4 (7.2)
**SF-12 physical composite score (0-100, <50 indicating worse physical health)**				
Baseline	10	42.0 (7.4)	9	41.7 (7.7)
Week 8	10	44.4 (7.5)	9	41.8 (12.5)
Week 12	10	47.9 (8.7)	9	39.4 (11.3)
Week 20	9	46.1 (5.0)	9	46.0 (8.2)
Week 52	9	47.1 (6.8)	8	43.6 (8.4)
**SF-12 mental composite score (0-100, <50 indicating worse mental health)**				

Baseline	10	38.5 (11.1)	9	37.7 (8.4)
Week 8	10	42.1 (12.1)	9	39.0 (9.3)
Week 12	10	41.1 (10.6)	9	39.3 (15.1)
Week 20	9	45.7 (8.8)	9	38.6 (10.9)
Week 52	9	43.0 (10.9)	8	43.0 (12.7)
**Penn-FPS-R (0 "no pain", 10 "total inability due to TN pain")**				
Baseline	10	4.1 (1.8)	9	5.5 (2.3)
Week 8	10	2.6 (2.1)	9	4.6 (3.1)
Week 12	10	3.1 (2.5)	9	4.4 (2.6)
Week 20	10	2.8 (2.7)	9	3.0 (2.0)
Week 52	10	3.7 (2.8)	9	3.1 (2.7)
**HADS – anxiety score (0-21, lower = better)**				
Baseline	10	9.4 (3.6)	9	8.4 (4.0)
Week 8	10	8.7 (2.5)	9	8.4 (4.0)
Week 12	10	9.5 (3.9)	9	9.1 (3.8)
Week 20	10	7.8 (3.3)	9	7.8 (1.9)
Week 52	10	8.5 (3.1)	8	7.4 (3.2)
**HADS – depression score (0-21, lower = better)**				
Baseline	10	6.8 (4.2)	9	8.4 (2.7)
Week 8	9	5.8 (3.8)	9	8.7 (3.7)
Week 12	10	6.3 (3.1)	9	9.6 (4.4)
Week 20	10	5.4 (3.5)	9	8.0 (3.0)
Week 52	10	6.9 (4.1)	8	6.3 (3.7)

Penn-FPS-R: Penn Facial Pain Scale-Revised; SES: Pain Perception Scale (Schmerzempfindungsskala); HADS: Hospital Anxiety and Depression Scale; NRS: numeric rating scale; 95% CI: 95% confidence interval;

Note: Patients in the intervention group received acupuncture from week 1 to week 8. Control group patients received acupuncture between week 12 and 20.

Table S3 a-d. Responder analysis

a) Responder reduction number of attacks ≥10% in week 8. OR (95%-CI) 1.88 (0.13, 48.48) p=0.65

Group	Yes	No
Acupuncture	7 (87.5%)	1 (12.5%)
Controls	7 (77.8%)	2 (22.2%)

b) Responder reduction intensity of attack pain ≥2 NRS in week 8. OR (95%-CI) 3.47 (0.34, 37.25) p=0.26

Group	Yes	No
Acupuncture	4 (50.0%)	4 (50.0%)
Controls	2 (22.2%)	7 (77.8%)

c) Responder reduction number of attacks ≥10% in week 12. OR (95%-CI) 0.91 (0.09, 9.94) p=0.94

Group	Yes	No
Acupuncture	4 (66.7%)	2 (33.3%)
Controls	6 (66.7%)	3 (33.3%)

d) Responder reduction intensity of attack pain ≥2 NRS points in week 12.

Group	Yes	No
Acupuncture	3 (33.3%)	6 (66.7%)
Controls	0 (0.0%)	9 (100.0%)

Table S4: STRICTA List (STandards for Reporting Interventions in Clinical Trials of Acupuncture- addendum to CONSORT point 5: intervention)

<u>**Item**</u>	<u>**Detail**</u>
**1. Acupuncture rationale**	1a) Style of acupuncture: Western medical
	1b) Reasoning for treatment provided: based on a consensus method with experts from two major acupuncture societies in Germany.
	1c) Treatment was varied: according to underlying TCM diagnosis
**2. Details of needling**	2a) Number of needle insertions per subject per session: starting from 6 – increasing to 17 per session- see methods section
	2b) Names of points: Mandatory points were GV20, and bilateral LI4, LV3, ST44, and GB20, with additional facial points tailored to the affected trigeminal branch: VI: GB14, EX-HN5 Taiyang – VII: GB41, ST3, ST4, ST6 – VIII: SI19, ST4, ST6. one to four additional facultative points were allowed at the discretion of the acupuncturists
	2c) Depth of insertion based on particular tissue level. SI 19 was punctured with opend mouth and 1 cun deep
	2d) Response sought (e.g. *de qi* or muscle twitch response): yes
	2e) Needle stimulation (e.g. manual, electrical): only to elicit DeQi once
	2f) Needle retention time: 25min
	2g) Needle type (0.25mm and 30mm or 0.3mm and 40mm, stainless steel, different manufacturers)
**3. Treatment regimen**	3a) Number of treatment sessions 10 treatments over 8 weeks
	3b) Frequency and duration of treatment sessions, first 2 weeks 2 sessions per week, then once per week
**4. Other components of treatment**	4a) Details of other interventions administered to the acupuncture group: none, routine medication
	4b) Setting and context of treatment: no further explanations or instructions, only inquire about side effects while settling of the patient for the next treatment.
**5. Practitioner background**	5) Description of participating acupuncturists: all had professional affiliation and more than 5 years in acupuncture practice
**6. Control or comparator interventions**	6a) Rationale for the control or comparator in the context of the research question: late entry in order to compare waitlist over 12 weeks and then gather data about replicability
	6b) Precise description of the control or comparator. Waitlist until week 12, then they received identical acupuncture regimen as described under 1-3.
